# A Series of Virtual Interventions for Chronic Lower Back Pain: A Feasibility Pilot Study for a Series of Personalized (N-of-1) Trials

**DOI:** 10.1162/99608f92.72cd8432

**Published:** 2022-09-08

**Authors:** Mark Butler, Stefani D’Angelo, Melissa Kaplan, Zarrin Tashnim, Danielle Miller, Heejoon Ahn, Louise Falzon, Andrew J. Dominello, Cirrus Foroughi, Thevaa Chandereng, Ken Cheung, Karina Davidson

**Affiliations:** 1 Feinstein Institutes for Medical Research, Northwell Health, Manhasset, New York, United States of America,; 2 Mailman School of Public Health, Columbia University, New York City, New York, United States of America,; 3 Donald and Barbara Zucker School of Medicine at Hofstra/Northwell, Northwell Health, Hempstead, New York, United States of America

**Keywords:** N-of-1, yoga, massage, personalized, personalized trial, pain

## Abstract

Chronic lower back pain (CLBP) affects 25% of U.S. adults and is associated with high costs due to physician visits and reduced productivity. Research shows that massage and yoga can be effective nonpharmacological treatments for CLBP, but the feasibility, scalability, individual treatment, and adverse-event heterogeneity of these treatments are unknown. The current study evaluated the feasibility and acceptability of a series of personalized (N-of-1) interventions for virtual delivery of massage and yoga or usual-care treatment for CLBP in 57 participants. We hypothesized that this study would provide valuable information about implementing a virtual, personalized platform for randomized controlled trials of personalized (N-of-1) interventions among individuals with CLBP. The study will do so by determining participants’ ratings of usability and satisfaction with the virtual, personalized intervention delivery system and, in the long term, identifying ways to integrate these personalized trials into patient care. Of the 57 participants enrolled, two withdrew from the study and were not eligible to receive the primary outcome assessment. Thirty-seven of the remaining 55 participants (67.3%) completed satisfaction surveys comprising the System Usability Scale (SUS) and items assessing satisfaction with the components of the personalized trial. Participants rated the usability of the personalized trial as excellent (average SUS score = 85.8), 95% were satisfied with the personalized trial overall, and 100% stated they would recommend the trial to others. These results suggest that personalized trials of massage and yoga are highly feasible and acceptable to participants with CLBP.

## 1. Introduction

Chronic pain, defined as pain lasting 12 weeks or more or persisting beyond the normal time for tissue healing (Institute of Medicine Committee on Advancing Pain Research Care Education, 2011) is one of the leading causes of disability. It can affect the physical and mental quality of life. Costs relating to chronic pain in the United States are roughly $560 to $635 billion in annual personal and health system expenditures. The prevalence of pharmacological treatment of chronic pain has led to concerns regarding the consequences of opioid treatment overuse, including overdose and death (Institute of Medicine Committee on Advancing Pain Research Care Education, 2011). As a result, the Centers for Disease Control and Prevention (CDC; Dowell et al., 2016) recommend non-opioid treatment over opioid therapy and note the need for additional primary research on alternative methods of managing chronic pain.

Of the various types of chronic pain, chronic low back pain (CLBP) is one of the most common reasons for physician visits. Approximately 25% of U.S. adults report having ongoing low back pain in the past 3 months (Deyo et al., 2006; (Qaseem et al., 2017). CLBP has been associated with $100 billion in health care costs and missed work or reduced productivity (Katz, 2006). To treat CLBP, the American College of Physicians recommends noninvasive and nonpharmacological treatments over pharmaceutical interventions (Qaseem et al., 2017). Nonpharmacological treatments for chronic pain include massage, exercise and physical therapy, mind-body practices, psychological therapies, interdisciplinary rehabilitation, mindfulness, osteopathic and spinal manipulation, acupuncture, physical modalities, and acupuncture (Skelly et al., 2018). While these interventions effectively reduce symptoms of chronic pain, identifying the most effective treatment for each patient is often difficult (van Tulder et al., 2000).

Both massage and yoga are nonpharmacological interventions that have been demonstrated to help treat CLBP. Previous research has shown that massage treatment for CLBP reduces pain compared to acupuncture or self-care Nahin et al., 2016) and relative to attention control (Little et al., 2008). Massage treatment may also lead to the use of fewer pain medications among persons with CLBP Nahin et al., 2016). Multiple types of massage interventions demonstrated effectiveness for CLBP pain reduction relative to usual care (Cherkin et al., 2011;. Further, massage interventions for CLBP had a low frequency of adverse events, albeit with heterogeneity in who experienced them (Cherkin et al., 2011; Nahin et al., 2016). Interventions using yoga for CLBP have demonstrated improved functioning and reduced levels of pain relative to controls (Wieland et al., 2017; (Williams et al., 2005). Yoga interventions for CLBP were associated with improvements in quality of life and reductions in pain intensity relative to exercise (Nambi et al., 2014). As with massage therapy, yoga therapy for CLBP had a low-but-heterogeneous frequency of adverse events (Williams et al., 2005). However, massage and yoga treatments for CLBP have not demonstrated consistent results among all patients and studies (Furlan et al., 2015; (Wieland et al., 2017); some persons with CLBP may benefit from or be harmed by these interventions more than others.

Though nonpharmacological interventions such as massage and yoga have demonstrated some efficacy, additional information is needed to determine which persons suffering from CLBP will most benefit from these interventions. Prior research has highlighted differences in adherence to treatment, patient population (e.g., variation in pain severity, and type of pain), and intervention dose may alter the effects of massage (Furlan et al., 2015; Imamura et al., 2008) and yoga (Wieland et al., 2017) for CLBP.

Personalized (N-of-1) trials are a patient-centered approach and single-case experimental research design (Vlaeyen et al., 2020) that provides essential clinical information for selecting the best treatments for individual patients. In a personalized trial design, individual patients are assessed using multiple crossover trials with continuous objective data collection over alternating time periods of one or more treatments and placebo therapies in randomized blocks Guyatt, 2016; Guyatt et al., 1990; . Personalized trials are specifically designed to help patients and their health care providers make treatment decisions informed by high-integrity, evidence-based information uniquely relevant to the important outcomes and values (Duan et al., 2013;. Prior series of personalized trials led participants to changes in treatment, cessation of treatment, or confirmation of the initial treatment (Duan et al., 2013; Guyatt, 2016; Guyatt et al., 1990; Joy et al., 2014; Larson, 2010). The National Pain Strategy report recommends that pain management be integrated, multimodal, interdisciplinary, evidence-based, and tailored to individual patient needs (Interagency Pain Research Coordinating Committee, 2015). Adhering to these recommendations, personalized trials are ideal in identifying pain-management strategies for CLBP. The randomized crossover design of personalized trials allows the same patient to receive multiple treatments for CLBP while continuously evaluating the effects of each treatment on multiple symptoms. In the case of CLBP, these symptoms could include pain, physical activity, and sleep. Once completed, patients and health care providers can use the information from the personalized trial to identify the treatment for CLBP that was most effective for each individual patient. This allows patients to receive the optimal treatment, improving outcomes and reducing overall costs caused by utilization of nonoptimal treatments (Scuffham et al., 2010).

The current study evaluates the feasibility and acceptability of a series of personalized interventions for virtual delivery of massage and yoga or usual-care treatment for CLBP in 57 participants. By utilizing new wearable technologies (such as Fitbit devices) and commercially available software platforms (such as Zeel), the current study allows continuous data collection and virtually conducted assessment. Further, virtual delivery of the intervention allows participants to receive treatment for CLBP in locations of their choosing and at times that are convenient for them. Results from this study will determine whether virtual delivery of these interventions is feasible and acceptable for patients with CLBP and allow clinicians to identify whether virtual delivery of massage and yoga can effectively treat CLBP.

## 2. Methods

### 2.1. Study Design

The study was a series of 57 randomized, personalized trials examining the effects of massage and yoga versus usual care on CLBP. The intervention was delivered virtually to participants in the tri-state area (i.e., New York, New Jersey, Connecticut) over 14 weeks. Participants were provided with a Fitbit Charge 3^™^ device and a Zeel account. Zeel is a technology platform that allows persons to book massage appointments with licensed and insured massage therapists. As part of this study, Zeel also ensured the availability of certified yoga instructors (from their Zeel@Work workplace wellness service) to participants for in-person, one-on-one yoga sessions. While accounting for travel, a commercially available platform such as Zeel allows for easy utilization and deployment of the intervention in multiple contexts and systems. Zeel only had access to the data provided directly by the participants.

The first 2 weeks of the study were a baseline assessment period. Participants could not book massage or yoga sessions using Zeel during this period and were discouraged from engaging in yoga or massage on their own.

During baseline assessment, each study participant was asked to engage in their usual methods of managing CLBP and wear their Fitbit device at all times, including during sleep. Participants were also asked to rate an ecological momentary assessment (EMA) of their pain, stress, and fatigue three times daily via text message. Each evening, they also answered a survey questionnaire assessing their back pain and pain management strategies. Each weekend, participants completed a longer survey measure asking them to reflect on their pain and pain management over the week. Participants were encouraged to wear their Fitbit devices day and night and were asked to sync their device with the Fitbit application on their phone at least every 2 days.

After completing the baseline period, participants were randomized into one of two arms with six 2-week yoga, massage, or usual-care treatment blocks. Zeel was used to allow participants to book one-hour sessions of Swedish massage and one-hour yoga sessions during the appropriate intervention blocks. In each week of the appropriate intervention block, participants were instructed to book two massage or two yoga sessions at least 48 hours apart. Study coordinators had access to booking confirmation receipts to ensure compliance with the protocol and to reeducate participants as necessary. Participants were discouraged from receiving additional massage or yoga sessions during treatment periods outside of those provided during the study. During usual-care periods, no treatment was provided to participants, who were discouraged from engaging in massage and yoga treatment independently. At the end of the 14 weeks, each participant was provided with a satisfaction survey and report containing their analyzed data. This report was sent within 3 months of study completion. After the satisfaction survey was completed, study coordinators reached out to each participant to interview them about their experience with the personalized trial. Study recruitment began in October 2019, and the study completion occurred in January 2021. Consolidated Standards of Reporting Trials (CONSORT), CONSORT Extension for Reporting N-of-1 Trials (CENT),, and CONSORT and SPIRIT Extension for RCTs Revised in Extenuating Circumstances (CONSERVE) reporting guidelines were utilized in this manuscript (Chan et al., 2013; (Orkin et al., 2021)Vohra et al., 2015).

### 2.2. Study Population

Participants in the current study included volunteer team members within the Northwell Health system who self-identify as having CLBP (defined as experiencing lower back pain for greater than or equal to 12 weeks). Prior research has demonstrated a high prevalence of CLBP among health care workers (Davis & Kotowski, 2015), especially nurses. The Northwell Health system offers a large potential pool of participants as it comprises approximately 77,000 employees. See Table 1 for inclusion and exclusion criteria.

### 2.3. Recruitment

Potential participants were primarily recruited via email messages sent out to all Northwell Health employees asking for persons with CLBP to participate in a personalized trial. Additional recruitment methods included referrals from Northwell Employee Health Services, social media, word of mouth, flyers distributed to Northwell Health facilities, and information presented at Northwell Health wellness events. Interested persons were directed to an online information screen with details about the pilot study and asked to complete an initial screening questionnaire containing study inclusion and exclusion criteria. This information was reviewed by study staff to determine participant eligibility before consent. If a potential participant was deemed ineligible or was waitlisted due to high demand, study staff notified the participant within 2 business days. If the participant was deemed eligible, the study staff sent them an email containing the electronic consent form and additional information within 2 business days.

### 2.4. Consent

Persons who were eligible to participate received an email from study staff with a link to access an electronic copy of the consent form as well as a short video explaining key details of the study protocol and consent form. A four-question screening measure assessed participant understanding of the protocol and consent process. Consent was obtained electronically, and a copy of the consent was mailed to the participant along with the study instructions and devices. Signed consent forms were stored electronically on a Health Insurance Portability and Accountability Act (HIPAA; Nosowsky & Giordano, 2006)–compliant, Northwell Health– approved shared drive accessible only to the institutional review board (IRB)–approved study staff. An example consent form can be found in the Supplemental Files.

Potential participants had the opportunity to choose from a list of start dates during their enrollment process. No more than 20 potential participants began their baseline period on the same day. Enrollment was ongoing until 57 participants were randomized to receive yoga and massage treatment periods after baseline.

### 2.5. Assignment of Interventions

The study statistician randomized participants to one of two treatment orders in blocks. Block randomization of participants in six blocks was created using a randomization website (Urbaniak & Plous, 1997). There were 29 participants randomized to the following order of 2-week treatment periods: Massage, Yoga, Usual Care, Usual Care, Yoga, and Massage. The remaining 28 participants were randomized to the following order of 2-week treatment periods: Usual Care, Yoga, Massage, Massage, Yoga, and Usual Care. These two treatment orders were utilized to simplify implementation of the pilot while also eliminating linear trends for analyses of treatment effects on pain.

### 2.6. Interventions

During massage treatment weeks, participants booked two one-hour Swedish massage sessions through Zeel. These massages were performed by a licensed and insured massage therapist employed by Zeel to minimize safety risks to the participant at the address specified by the participant when booking the session. In addition, massage sessions were scheduled at least 48 hours apart from one another in a single Monday-to-Sunday week.

Thus, participants had the option of booking up to four massage sessions, or 2 weeks of treatment, at a time. Participants booked massages by logging into the study account provided to them at www.Zeel.com or by downloading the Zeel app.

During yoga treatment weeks, participants received a text message with a link to a form to sign up for up to two one-hour Viniyoga sessions a week for one treatment period (i.e., 2 weeks or four total yoga sessions). Participants were instructed to schedule these two yoga sessions at least 48 hours apart from another within the same Monday-to-Sunday week. The yoga sessions were delivered by a certified yoga instructor employed by Zeel at the address specified by the participant on their scheduling form. In addition, in-person, one-on-one treatment sessions with a yoga instructor were provided to minimize safety risks to the participant.

During usual-care treatment periods, participants were asked to refrain from participating in any massage or yoga sessions and manage their CLBP using the methods they usually would.

### 2.7. Participant Timeline

**Figure 1.**
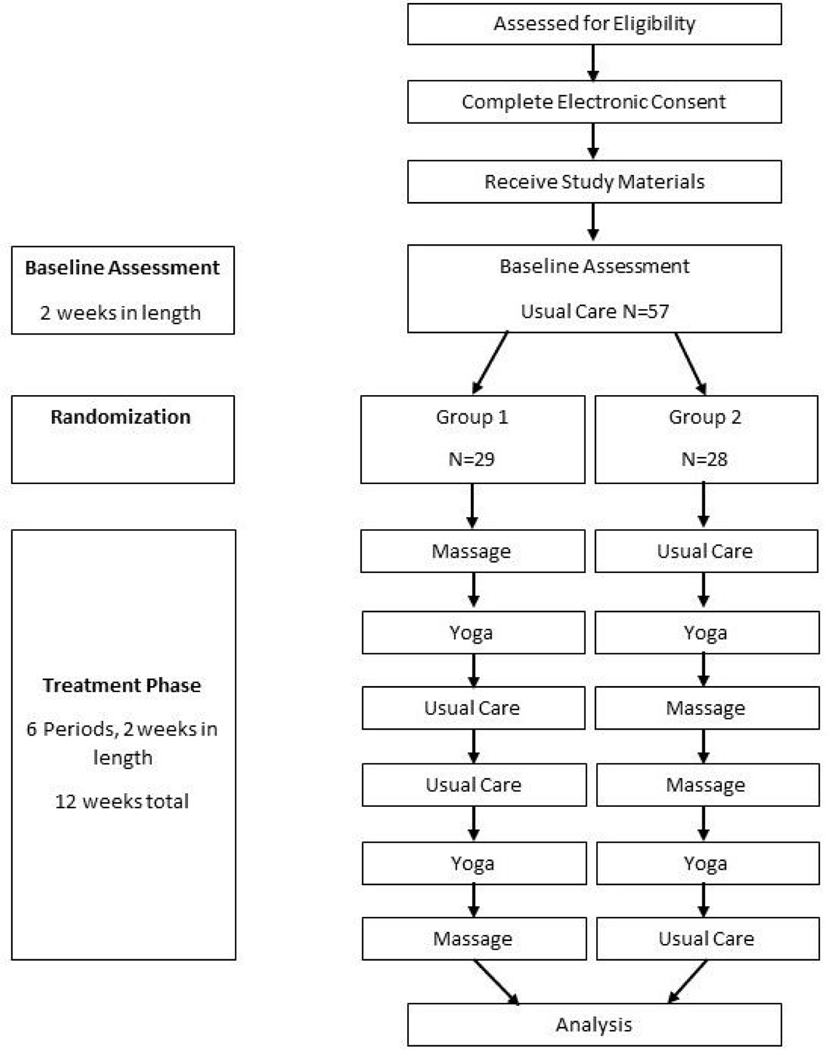
Participant timeline.

Figure 1 illustrates the participant timeline.

### 2.8. Adherence

Participant adherence to the protocol was assessed during the first 14 days of the baseline assessment period. During baseline assessment, study staff reviewed participant adherence to wearing their Fitbit, EMA measure completion, and survey responses. Participants wearing the Fitbit more than 12 hours per day and while sleeping and those who responded to EMA and survey measures were defined as adherent. During the 14 days of the baseline period, participants who did not achieve a minimum of 80% adherence to Fitbit wear and study measures were withdrawn from the study. Participants maintaining 80% adherence or greater continued baseline assessment and were randomized to the treatment phase.

Several methods were used to encourage adherence throughout the study. For example, participants had short education videos made available to them, provided protocol reminders via text message, and encouraged them to contact study staff with concerns by phone or email.

### 2.9. Extenuating Circumstances Due to COVID-19

The current trial was in the middle of intervention delivery during the beginning of the coronavirus disease 2019 (COVID-19) pandemic. New York State enacted the “New York State on PAUSE” executive order beginning March 22, 2020. In addition to the state-mandated lockdown, study investigators and staff were concerned with causing a potential increased burden on a participant population comprising frontline health care workers. As a result, the trial paused enrollment and delivery of massage and yoga interventions between March 21, 2020, and August 3, 2020. Data collection was also paused during this period.

**Figure 2.**
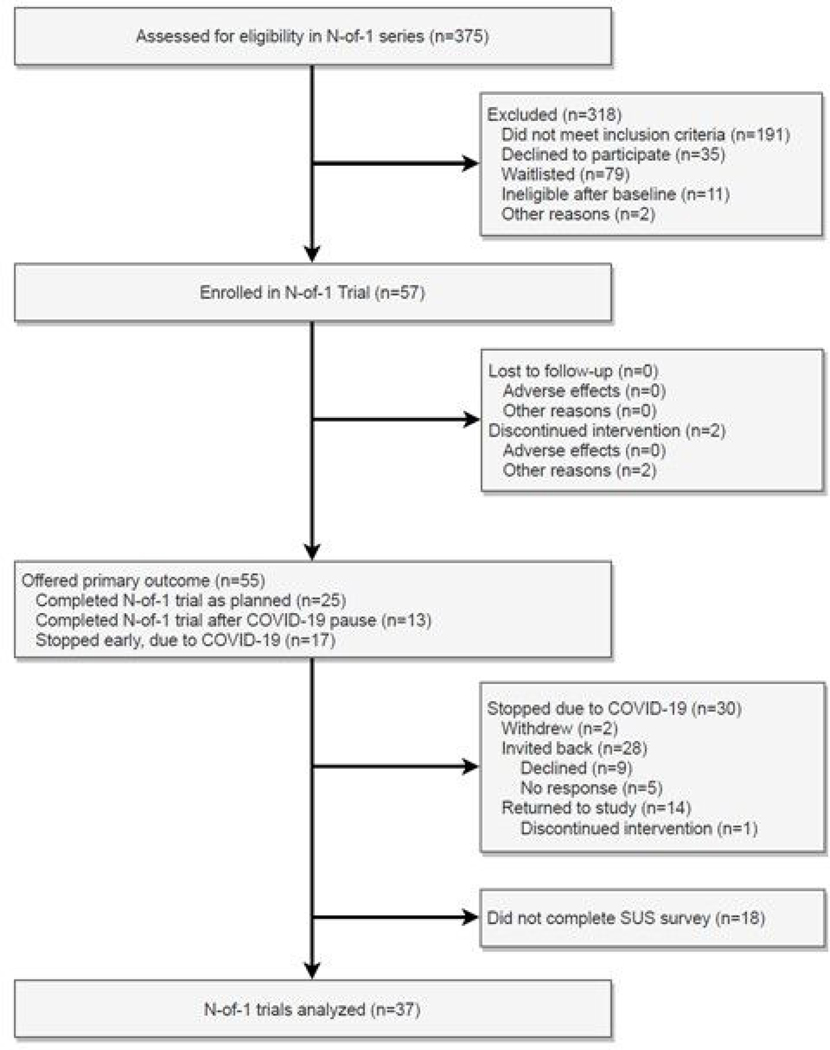
CONSORT flow diagram. SUS = System Usability Scale.

Modifications to the protocol to address these extenuating circumstances included a temporary pause in delivering massage and yoga interventions and corresponding data collection. In addition, at the request of the study’s Data Safety Monitoring Board (DSMB), the satisfaction survey administered at trial completion was modified to ask participants how they managed their CLBP during the study pause. Once the lockdown ended and protocol activities were permitted to resume at the study institution, affected participants were invited to resume the intervention if they wished to continue. A total of 25 participants completed data collection before the study pause (Figure 2). An additional 13 participants chose to return to the study after the pause to complete the intervention as planned. Seventeen participants did not resume the intervention following the pause in the study. Details about this can be found in the CONSORT diagram in Figure 2. Recruitment was paused at 57 participants due to concerns about another COVID-19 surge.

Modifications to the study protocol based on extenuating circumstances included the pause in the intervention, changes in the intervention for participants who chose not to resume the trial following the pause, change in the number of participants enrolled, and the addition of a single measure to the participant satisfaction survey administered at trial completion. Post-lockdown massage and yoga interventions were slightly modified to increase infection control measures as determined by the study’s commercial vendor, Zeel (e.g., use of outdoor locations). Due to the extenuating circumstances, the study team chose to create and deliver participant reports to those who chose not to return to study participation, using data that had been collected before the study pause. No modifications were made to the study introduction, methods, or results.

Modifications to the protocol were planned by the study’s principal investigator, approved by the Northwell Health IRB and study DSMB, and implemented by the principal investigator, study coordinator, and research team. No interim data were collected to determine modifications to the trial to address extenuating circumstances. However, as recommended for trials affected by extenuating circumstances (Orkin et al., 2021), a CONSORT-CONSERVE Checklist was completed and is included the Supplemental Files.

## 3. Outcomes

### 3.1. System Usability Scale

The primary outcome of the current study is the System Usability Scale (SUS; Brooke, 1996), a validated 10-item questionnaire that asks users to rate the usability of systems. The system usability scale assesses multiple aspects of systems including effectiveness and efficiency. Each item is rated on a Likert scale from Strongly Disagree (1) to Strongly Agree (5). Individual item scores are reduced by 1 (odd-numbered questions) or subtracted from 5 (even-numbered questions), then multiplied by 2.5 and summed to generate a total score ranging from 0 to 100 with higher scores indicating a greater level of usability. Participants completed the SUS after completion of treatment. This measure has been utilized and validated in multiple contexts (Brooke, 2013; Lewis, 2018). The SUS can be interpreted by comparing scores to other comparable and established systems. We will compare SUS scores in the current trial to other virtual interventions. For the current trial, the SUS will assess the feasibility of the virtual intervention.

### 3.2. Secondary Outcomes

#### 3.2.1. Satisfaction and Feedback Survey

Patient satisfaction with the trial was assessed using a satisfaction and feedback survey administered upon completion of the treatment. The survey assessed participant satisfaction with elements of the trial, including the onboarding process, the consenting process, the Fitbit device, the personalized trial design, assessment measures, and feedback received from the study team. Participant satisfaction with the interventions (both massage and yoga) and the Zeel application/website was also assessed. The survey included statements such as “How satisfied were you with your personalized trial of yoga and massage for chronic lower back pain?” In addition, participants were asked to rate their satisfaction on a scale of 1 (“Not very satisfied”) to 5 (“Very satisfied”). There were two segments of the satisfaction survey administered to participants asking about Elements of a Personalized Trial as well as Satisfaction with Components of the Trial. For the current trial, satisfaction will be used to assess the acceptability of the current trial.

#### 3.2.2. Effectiveness Outcomes

Effectiveness outcomes in the current study include the Patient-Reported Outcomes Measurement Information System (PROMIS) pain scale pain intensity ratings, PROMIS pain scale pain interference ratings, EMA self-reported pain ratings, EMA self-reported fatigue ratings, EMA self-reported stress ratings, self-reported use of pain medication, Fitbit device–recorded daily steps, and Fitbit device–recorded nightly sleep duration. The PROMIS pain (Amtmann et al., 2010; Revicki et al., 2009) scales, version 1.0, were used to measure the intensity of pain symptoms (Pain Intensity 3a Fixed Length Short Form) and interference (Pain Intensity 4a Fixed Length Short From) with daily life due to pain symptoms. Both pain intensity and pain interference measures were slightly modified to ask participants to reflect on pain over the past 24 hours rather than the past 7 days. All items are rated on a scale of 1 to 5, with higher scores indicating higher pain intensity or interference levels. The reliability and validity of the PROMIS-Pain Interference scales have been well supported (Chen et al., 2018). Daily self-reported pain, fatigue, and stress ratings were assessed via EMA using a measure adapted from the Numeric Pain Rating Scale. These assessment measures are single-item assessments administered three times daily via text message asking participants to rate their pain, fatigue, and stress in the current moment on a scale of 0 to 10. The timing of the text messages was randomized throughout participants’ self-reported wake hours. For pain, ratings of 0 indicate no pain, with scores of 1–3, 4–6, and 7– 10 respectively indicating mild, moderate, and severe pain. Interpretations of scores remain the same for fatigue and stress. Self-reported use of pain medication was assessed by surveys administered at the end of each day and week. Participants were asked to report the type of over-the-counter or prescription-strength pain medication they used for their back pain, the dose of the medication, and the frequency of medication use. Daily Steps and Nightly Sleep Duration were assessed using non–Near Field Communication (NFC), Fitbit Charge 3^™^ devices. The current trial evaluates the feasibility and acceptability of this series of personalized interventions for virtual delivery of massage and yoga or usual-care treatment for CLBP. Effectiveness outcomes will be analyzed and reported upon in a separate manuscript.

## 4. Analysis

### 4.1. Sample Size Calculation

The sample size of 60 participants was chosen to ensure a sufficient number of patients to obtain a preliminary assessment of the feasibility and acceptability of this series of personalized trials of massage and yoga for CLBP. The numbers of questionnaires and treatment repetitions per trial were based on expert recommendations by a statistician and estimations about maximal duration of the trial to maintain patient engagement. With *n* = 60, using a one-sample binomial test at 2.5% significance one-sided, this would have given the current study approximately 90% power if the trial completion rate was 70%. Unfortunately, the sample size was reduced to 57 participants due to COVID-19 concerns. The numbers of assessment measures and treatment repetitions per trial were based on expert recommendations and estimations about the maximal duration of the trial to maintain patient engagement.

### 4.2. Analyses

The primary analysis focused on participant usability. Usability ratings from all enrolled participants on the SUS were averaged together to obtain an overall usability score for the study. This average score was then compared to established standards of usability in the SUS literature to determine the relative usability of the intervention protocol. Means, standard deviations and frequencies for participant responses to the satisfaction survey were also examined. As the primary goal of this study is to examine the feasibility and acceptability of this series of personalized trials, no outcomes relating to effectiveness were analyzed.

## 5. Results

### 5.1. Enrollment and Sample Characteristics

Of the 57 participants enrolled in the trial, 25 participants completed the trial as planned. Two participants withdrew from the study prior to March 21, 2020. Due to extenuating circumstances related to COVID-19, the intervention was paused from March 21, 2020, through August 3, 2020, for 30 participants (Figure 2). During the pause in the intervention, another two individuals withdrew due to COVID-19 concerns and 28 were offered the opportunity to continue the intervention. Of this group, 14 individuals declined/did not respond to the request and 14 resumed the intervention. As 55 of the enrolled 57 participants received all or part of the intervention, these individuals were sent the SUS and a satisfaction survey to evaluate the feasibility and acceptability of the trial. Completed survey measures of the SUS and satisfaction were received for 37 of the 55 (67.3%) participants who were offered the primary outcome measures.

The sample of 55 participants who received the primary outcome was composed of 75% (*N* = 41) women, had a mean age of 42.6 (*SD* = 13.0), was 58% (*N* = 32) White, and 15% (*N* = 8) Hispanic/Latino (Table 2). Participant characteristics did not differ between the two treatment orders (Table 2). Table A1 shows participant responses to the survey by whether their intervention was paused due to COVID-19. No differences in characteristics between participants who responded to the primary outcome measure (*N* = 37, 67.3%) and participants who did not (*N* = 18, 32.7%; Table A1) were observed. Further, no differences in characteristics between participants who had a pause in the intervention due to COVID-19 (*N* = 30, 54.5%) and participants who completed the trial as planned (*N* = 25, 45.5%) were observed (Table A2).

Adherence to the survey measures, Fitbit device use, and to the treatment were relatively high over the duration of the study. In the sample of participants who received the primary outcome (*N* = 55), the mean participant adherence to survey completion (including both EMA and other survey assessments) was 81.2%. Participants were adherent to Fitbit device use (defined as wearing the device 12 or more hours per day) for an average of 89.4% of days across the study duration. Participants also were adherent to treatment (defined as completing two treatment sessions per week) for an average of 86.5% of weeks during treatment blocks.

### 5.2. System Usability Scale (SUS)

**Figure 3.**
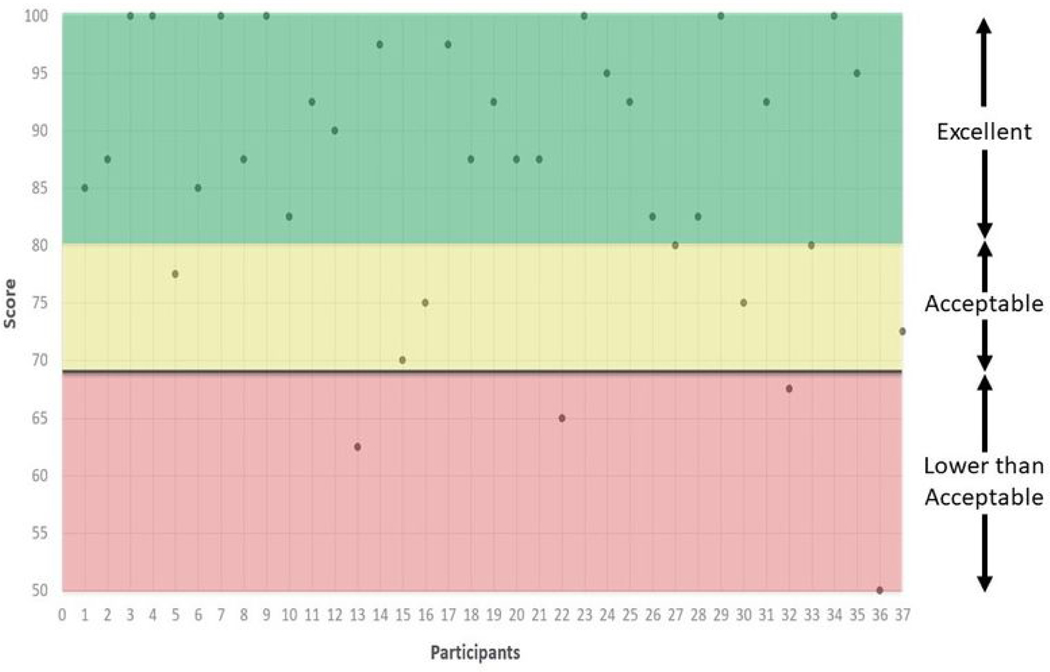
Scores on the System Usability Scale (SUS) by participant.

Participants who responded to the survey reported high levels of usability from the SUS [Mean (*SD*) = 85.8(12.3)]. This average SUS score indicates that the trial is an excellent system (SUS total score≥ 85; Bangor et al., 2008). Individual scores indicate that most participants rated the trial as acceptable (SUS total score≥ 70; Bangor et al., 2008) or better. Seven participants (19%) responded with the highest possible score for usability. Only four participants (11%) rated the trial as having lower-than-average usability (Figure 3). Participant scores on the SUS are reported in order of enrollment in Figure 3.

**Figure 4.**
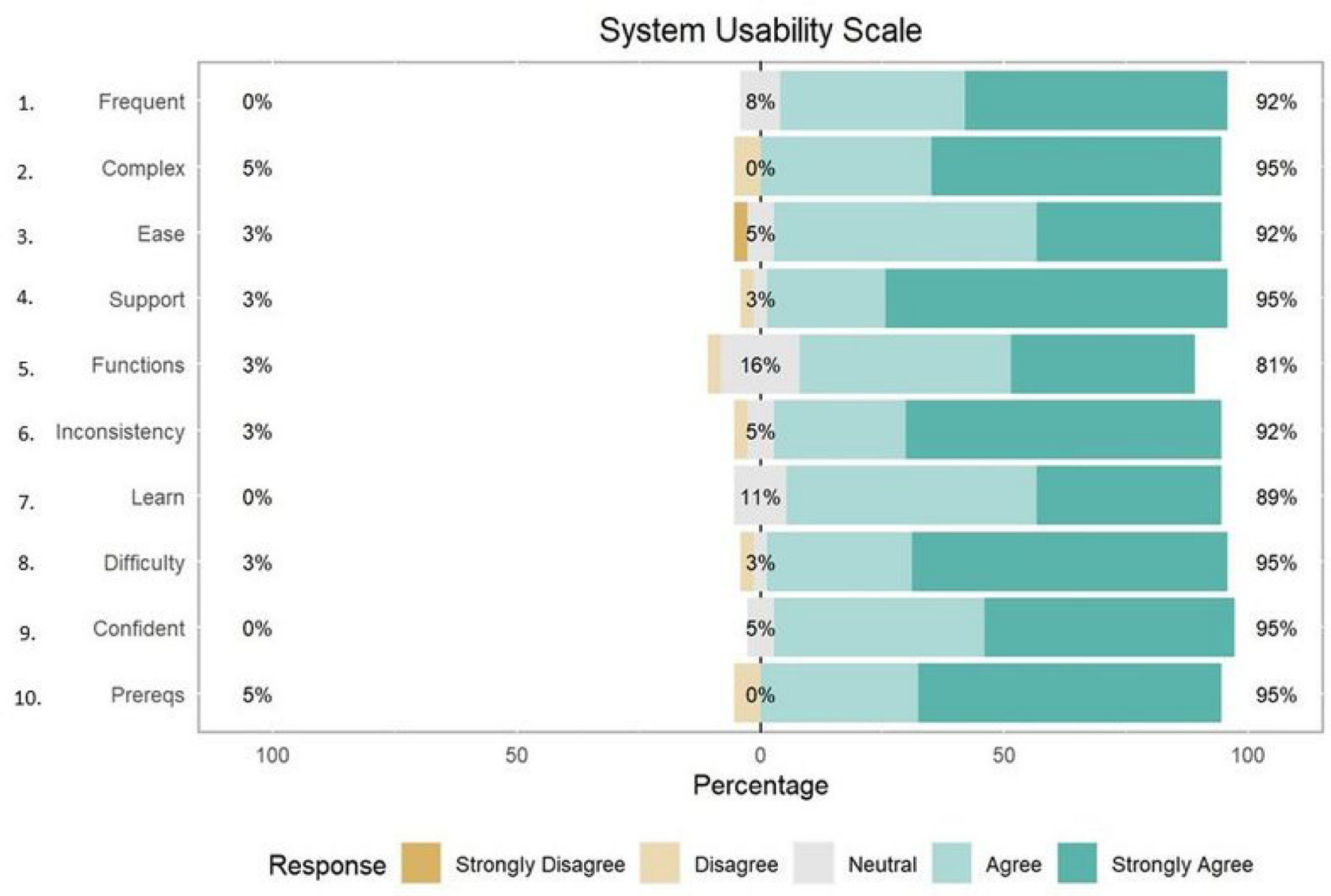
Distribution of responses for individual items on the System Usability Scale (SUS).

Average responses to individual items on the SUS (Table 3) ranged from 4.16 (*SD* = 0.80) for the item “I found the various functions in this system were well integrated” to 4.62 (*SD* = 0.68) for the item “I do not think that I would need the support of a technical person to be able to use this system.” The distribution of responses to individual items on the SUS can be found in Figure 4.

To determine whether the pause in enrollment due to COVID-19 influenced participant feasibility ratings, we compared SUS scores between participants who had their protocol paused (*N* = 30) and participants who did not (*N* = 25). Scores on the SUS were higher in the group without a pause [Mean (*SD*) = 87.4 (11.5)] than in the group who were paused [Mean (*SD*) = 83.5 (13.6)] but were not significantly different using an independent samples *t* test (*p* = .35).

### 5.3. Participant Satisfaction and Attitudes

**Figure 5.**
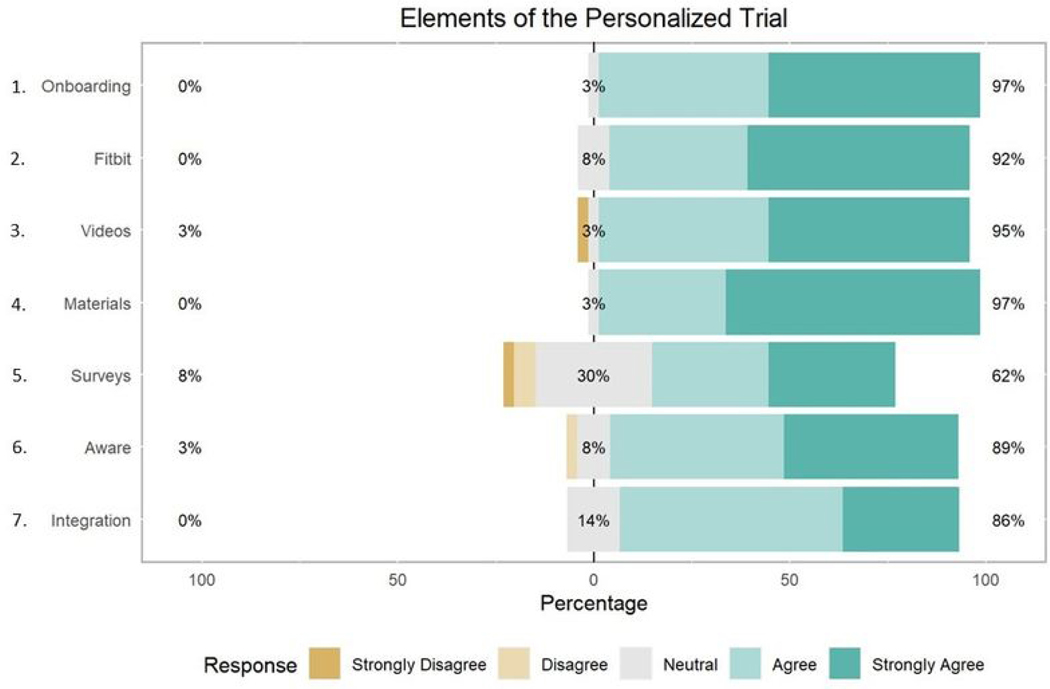
Participant satisfaction with elements of the personalized trial.

**Figure 6.**
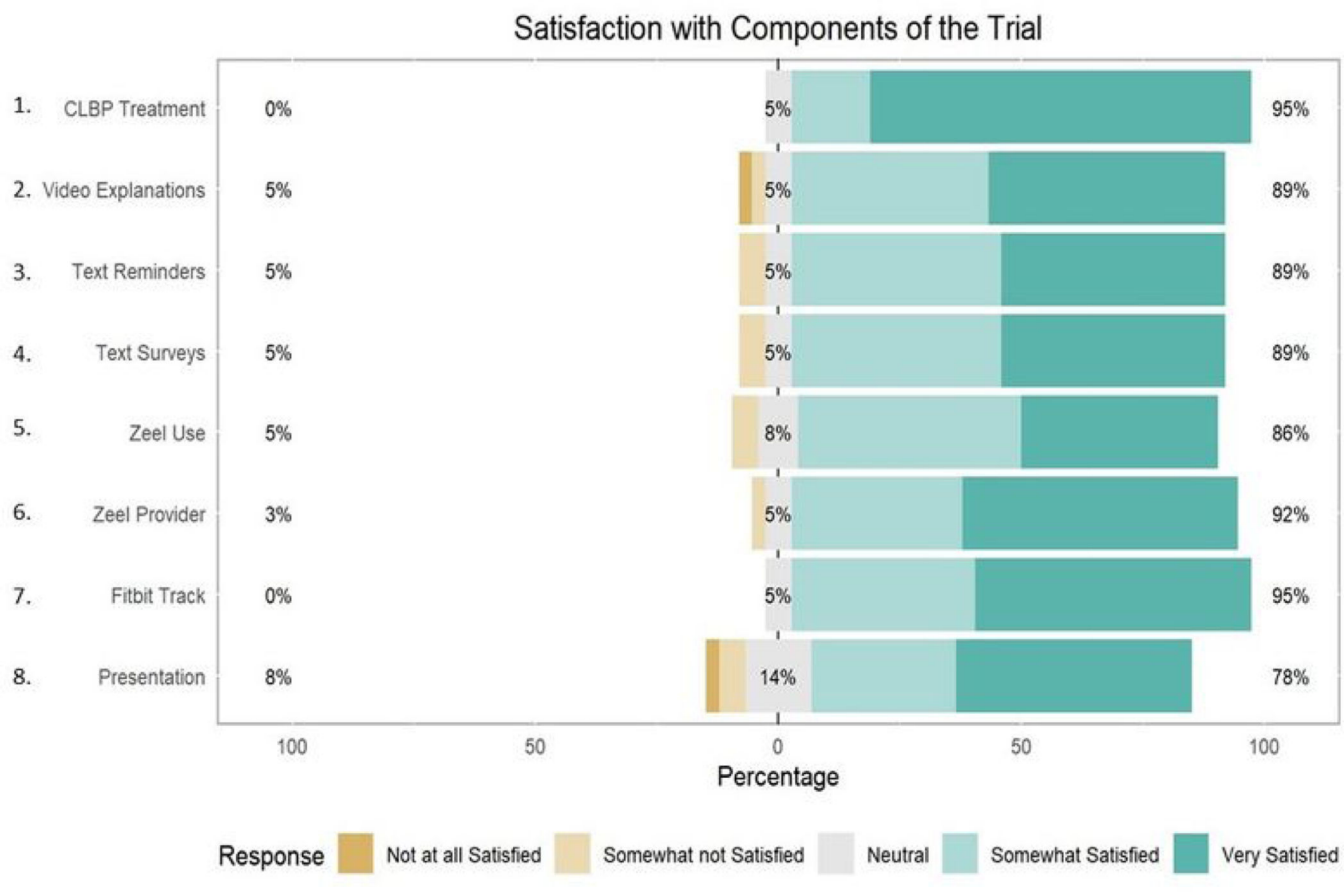
Participant satisfaction with components of the trial. CLBP = chronic lower back pain.

Participants demonstrated high levels of satisfaction with elements of the personalized trial, including the consenting process, Fitbit device, study materials, and text-message interventions, with average responses ranging from 3.8 to 4.7, indicating satisfaction (Table 4). Respondents were least positive about the daily text messages and surveys in the trial, with 62% (*N* = 23) rating them favorably and 8% (*N* = 3) rating them unfavorably (Figure 5). Participants were most positive about the trial overall, with 95% (*N* = 35) of participants rating it favorably and 5% (*N* = 2) rating it neutral (Figure 6). In addition, 97% (*N* = 36) of participants agreed that the onboarding process was easy to follow and that materials they received by mail were easy to use (Figure 5). Eighty-six percent (*N* = 32) of participants felt the trial was easy to integrate into their daily routine (Figure 5). Most participants (*N* = 32, 86%) were satisfied with Zeel as the provider for massage and yoga.

All 37 participants who completed the satisfaction survey stated that they would strongly recommend the trial (94.6%) or would recommend the trial “a little bit” (5.4%) to other individuals with lower back pain (Table 5). Most of the participants stated in the survey that the study was helpful in respect to their symptoms of lower back pain. Seven individuals responded that their participation in the study was somewhat helpful, while 18 participants said it was very helpful. Ten participants stated that it was extremely helpful. Only two participants stated that their participation in the study was a little helpful (Table 5). Participants were also asked which treatment they would utilize for CLBP and were allowed to select more than one option. Based on Table 5, 24 (64.9%) individuals stated they would continue the massage, 27 (73.0%) participants would continue yoga, 1 (2.7%) participant would continue neither, and 4(10.8%) participants would continue an alternative method to manage their back pain. The participants who had a pause in the trial due to COVID-19 managed their CLBP with various methods, with the most frequently endorsed being over-the-counter pain medications and self-directed yoga (Table 5).

To determine whether the pause in enrollment due to COVID-19 influenced participant usability ratings, we compared scores on satisfaction items between participants who had their protocol paused (*N* = 30) and participants who did not (*N* = 25). Scores on the satisfaction measures were usually higher in the group without a pause, but no satisfaction scores differed between groups based on an independent samples *t* tests (Table A4).

## 6. Discussion

### 6.1. Discussion of the Current Trial

The results from the current trial show positive evaluations of virtually delivered personalized N-of-1 massage and yoga interventions for CLBP. The SUS scores obtained for the current trial indicate excellent levels of usability and were higher than those for several virtual interventions targeting unsafe medication use (Holden et al., 2020), diabetes (Balsa et al., 2020; (Fu et al., 2020; Ye et al., 2018), obesity (Lee et al., 2019), stroke rehabilitation (Levac et al., 2016), and rehabilitation of respiratory failure survivors (Parker et al., 2020). Ratings of the SUS in the current trial were comparable to both a virtual intervention to reduce sedentary behavior after cancer surgery (Low et al., 2020) and an app-based intervention for depression (Fuller-Tyszkiewicz et al., 2018). Thus, the current trial has comparable or superior levels of usability relative to other virtually delivered interventions in the literature. Analysis of the individual items of the SUS indicates that participants stated they believed the trial program was easy to use, consistent, well integrated, and not overly complex. This supports that this series of virtually delivered personalized N-of-1 trials of massage and yoga interventions for CLBP is highly feasible.

Further, participants reported that they did not need help to engage with the trial. This suggests that the personalized N-of-1 methodology utilized in the current study could be integrated into clinical practice with modest support. Sixteen percent of those who responded provided a neutral score to question 5 (“I found the various functions in this system were well integrated”), and this item was the lowest individual SUS item in terms of overall participant satisfaction. During this study, participants were required to interface with three different applications (Fitbit, Zeel, and REDCap) to satisfy all treatment delivery components and data collection. As this study aimed to determine the initial feasibility and acceptability of an N-of-1 platform, investment of time or additional resources to integrate study components was skipped in favor of a more agile deployment. Future personalized trials should focus on integrating various components of the N-of-1 experience (e.g., Fitbit device, commercial service booking) into one platform to reduce the burden on participants.

In assessing participant satisfaction with elements and components of the personalized trial, we found that participants were generally satisfied with all aspects of the trial, including phases of the trial (e.g., onboarding), devices (e.g., the Fitbit wearable device), and treatments through Zeel. The lowest levels of satisfaction were with the text message and survey assessments used in the study. Though most participants still rated the assessment favorably (62%), future personalized trials could benefit from altering the frequency and delivery method of assessments to maximize participant satisfaction.

Further, all respondents to the satisfaction survey in the current study stated that they would recommend the personalized N-of-1 trial to others and found the trial to be at least a little helpful. This indicates that, overall, participants found the trial to be useful and beneficial. These findings suggest that this series of virtually delivered personalized N-of-1 massage and yoga interventions is highly acceptable to participants with CLBP.

### 6.2. Strengths and Limitations

The current study has several notable strengths. First, using new wearable technologies (such as Fitbit devices), virtual assessment measures, and the commercially available Zeel software platform means that the current study’s design is easy to replicate and can be scaled quickly to recruit larger samples. Second, feasibility and participant satisfaction with the trial was assessed using multiple metrics, including the SUS, a validated measure of usability utilized to evaluate other virtual interventions. This measure was supplemented with a satisfaction survey designed to assess participant attitudes about specific components of the current trial. The use of the SUS and a tailored satisfaction survey allows for comparison of the current trial with other similar studies while also evaluating aspects unique to the current trial.

Limitations include the current study’s sample being recruited from a single health care system. Persons working within a health system may have different evaluations of virtual, personalized interventions than persons from the general public. Additionally, 18 participants failed to complete the SUS or satisfaction measures evaluating the trial. While the response of the individuals who did complete the survey measures was positive, knowing the experience of the participants who did not respond to the evaluations is impossible; the current trial may have been less acceptable to these individuals. If this is the case, the true levels of participant acceptability may be lower than those reported in the current trial. While many potential barriers have been identified for lack of participation in satisfaction surveys (e.g., age, sex, insurance status, language barriers, cognitive deficits; Boscardin & Gonzales, 2013; Gayet-Ageron et al., 2011; Tyser et al., 2016), nonresponders and responders had similar demographic characteristics in the current study (Table A1). Prior studies examining treatment satisfaction surveys have found that nonresponders tend to have lower levels of satisfaction with treatment; however, the differences in satisfaction between responders and nonresponders have been found to have small magnitudes (Lasek et al., 1997). Given the high levels of satisfaction with the components of the trial and the overall trial, it is unlikely that nonresponders would rate the trial as much less feasible and acceptable than responders. This prior research suggests that the lack of data for 18 participants in the current study is unlikely to dramatically change our findings. Finally, some participants experienced a pause in the trial due to COVID-19. Thirty participants did not receive the intervention as planned due to a pause in the trial caused by extraneous circumstances related to COVID-19. It is possible that this change in the presentation of the intervention may have altered how participants rated the feasibility and acceptability of the study design. However, comparisons between these groups show that individuals who paused the intervention had comparable ratings of feasibility and acceptability to those who did not.

### 6.3. Conclusion

In conclusion, the current findings illustrate that a series of personalized interventions for virtual delivery of massage and yoga are both feasible (based on high levels of usability on the SUS) and acceptable (based on high levels of satisfaction) in participants with CLBP. However, additional work will be required to examine the secondary outcomes from the current series of personalized trials in order to determine whether massage and yoga therapy had a heterogeneous pain reduction response and the extent of such heterogeneity. Acceptance to the participant-facing report is further discussed in the article “Personalized Feedback for Personalized Trials: Construction of Summary Reports for Participants in a Series of Personalized Trials for Chronic Lower Back Pain,” which is also located within this special issue (D’Angelo et al., 2022).

## Supplementary Material

Supplementary Material

## Figures and Tables

**Table 1.** Study inclusion and exclusion criteria.

Inclusion Criteria	Exclusion Criteria
• Participants who met the following criteria were included in the study: At least 18 years of age • Fluent in English • Experiencing symptoms of lower back pain for 12 or more weeks • Experiencing a self-reported pain intensity ≥ 8 on the Patient-Reported Outcomes Measurement Information System (PROMIS) Pain Interference 8a short-form scale • Able to receive interventions (two times per week between 8:00 a.m. and 10:00 p.m. Monday through Sunday) • Possessing a smartphone capable of receiving text messages • Possessing an e-mail account that can be regularly accessed	Persons who met the following criteria were excluded: • Pregnant women • Current opioid users • Having a history of spinal surgery • Experiencing complex back pain (e.g., sciatica, spinal stenosis, or other pre-existing condition) • Having had a previous diagnosis of a serious mental health condition or psychiatric disorder • Having had a previous diagnosis of opioid use disorder or treatment for any substance use disorder • Having been previously advised that yoga or massage is unsafe for their condition • Being limited by physical activity restrictions • Planning surgery or procedures within six months of recruitment • Planning travel outside the US within treatment period timeframe • Weighing greater than or equal to 500 pounds

**Table 2.** Descriptive characteristics.

Variable		Total Sample *N* = 55	TreatmentOrder 1* *N* = 28	Treatment Order 2* *N* = 27	*p* value
Age; Mean (*SD*)		42.6 (13.0)	41.6 (13.4)	43.6 (12.7)	.538
Sex; *N* (%)	Female	41 (74.5%)	23 (82.1%)	18 (66.7%)	.314
	Male	14 (25.5%)	5 (17.9%)	9 (33.3%)	
Race; *N* (%)	Asian	11 (20.0%)	4 (14.3%)	7 (25.9%)	.861
	Black	6 (10.9%)	3 (10.7%)	3 (11.1%)	
	Mixed	2 (3.6%)	1 (3.6%)	1 (3.7%)	
	Other	4 (7.3%)	2 (7.1%)	2 (7.4%)	
	White	32 (58.2%)	18 (64.3%)	14 (51.9%)	
Ethnicity; *N* (%)	Hispanic	8 (14.5%)	4 (14.3%)	4 (14.8%)	.999
	Non-Hispanic	47 (85.4%)	24 (85.7%)	23 (85.2%)	

* Treatment Order 1: Massage, Yoga, Usual Care, Usual Care, Yoga, Massage; Treatment Order 2: Usual Care, Yoga, Massage, Massage, Yoga, Usual Care.

*Note. P* values for comparisons of participant characteristics between treatment orders are obtained from independent samples *t* tests for continuous variables and Pearson chi-squared tests for categorical variables.

**Table 3.** Descriptive statistics for the system usability scale (SUS).

Measure		Mean (*SD*)	Range
System Usability Scale Overall Score		85.81 (12.35)	[50, 100]
System Usability Scale Individual Items			
Item	1. I think that I would like to use this system frequently.	4.46 (0.65)	[3, 5]
	2. I did not find the system unnecessarily complex.*	4.49 (0.77)	[2, 5]
	3. I thought the system was easy to use.	4.24 (0.80)	[1, 5]
	4. I do not think that I would need the support of a technical person to be able to use this system.*	4.62 (0.68)	[2, 5]
	5. I found the various functions in this system were well integrated.	4.16 (0.80)	[2, 5]
	6. I did not think there was too much inconsistency in this system.*	4.54 (0.73)	[2, 5]
	7. I would imagine that most people would learn to use this system very quickly.	4.27 (0.65)	[3, 5]
	8. I did not find the system very cumbersome or awkward to use.*	4.57 (0.69)	[2, 5]
	9. I felt very confident using the system.	4.46 (0.61)	[3, 5]
	10. I did not need to learn a lot of things before I could get going with this system.*	4.51 (0.77)	[2, 5]

* Items were initially reverse coded but have been recoded to be on the same scale as other items. The text of these questions has been revised from the original items to reduce confusion.

*Note.* Questions rated on a 5-point Likert scale from 1 “Strongly Disagree” to 5 “Strongly Agree.”

**Table 4.** Descriptive statistics for satisfaction measures.

Measure		Mean (*SD*)	Range
Elements of the Personalized Trial*			
Items	1. I found the onboarding process (from the initial survey to getting my materials) for my personalized trial straightforward and easy to follow.	4.51 (0.56)	[3, 5]
	2. I think my Fitbit Charge 3 was easy to use.	4.49 (0.65)	[3, 5]
	3. The informational videos helped me understand how to participate in this study.	4.41 (0.80)	[1, 5]
	4. The materials I received in the mail were clear and easy to use.	4.62 (0.55)	[3, 5]
	5. I enjoyed receiving daily text message prompts and surveys on my cell phone.	3.84 (1.04)	[1, 5]
	6. I felt like I knew what was coming next in my personalized trial.	4.31 (0.75)	[2, 5]
	7. My personalized trial was easy to integrate into my daily routine.	4.16 (0.65)	[3, 5]
Satisfaction with Components of the Trial**			
Items	1. Your personalized trial of yoga and massage for chronic lower back pain.	4.73 (0.56)	[3, 5]
	2. Video explanations and demonstrations of study devices and procedures.	4.30 (0.91)	[1, 5]
	3. Text messaging for reminders.	4.30 (0.81)	[2, 5]
	4. Text messaging for survey questions.	4.30 (0.81)	[2, 5]
	5. Use of the Zeel application/website and survey for at-home yoga and massage booking.	4.22 (0.82)	[2, 5]
	6. Zeel as a provider for your massage therapist or yoga instructor.	4.46 (0.73)	[2, 5]
	7. Use of the Fitbit Charge 3 to track your activity and sleep.	4.51 (0.61)	[3, 5]
	8. Presentation of your results.	4.16 (1.04)	[1, 5]

* Questions rated on a 5-point Likert scale from 1 “Strongly Disagree” to 5 “Strongly Agree.”

** Questions rated on a 5-point Likert scale from 1 “Not at all Satisfied” to 5 “Very Satisfied.”

**Table 5.** Participant ratings of the helpfulness of the N-of-1 trial.

Response	*N*	%
How much would you recommend this trial of yoga and massage therapy to other persons with symptoms of lower back pain?		
I would not recommend	0	0.0
I would recommend a little bit	2	5.4
I would strongly recommend	35	94.6
Overall, how helpful was your participation in this study with respect to your symptoms of lower back pain?		
Not at all helpful	0	0.0
A little bit helpful	2	5.4
Somewhat helpful	7	18.9
Very much helpful	18	48.7
Extremely helpful	10	27.0
During the stay-at-home order from March – June, how did you manage your back pain?		
Yoga with virtual programs (i.e., mobile apps, online classes, YouTube videos, etc.)	7	18.9
Yoga on your own (previous experience)	9	24.3
Massage devices	2	5.4
Over-the-counter pain medications	10	27.0
Other	9	24.3
Now that you have completed this study and reviewed your results, will you be continuing either massage or yoga to manage your back pain? Please select all that apply.		
Massage	24	64.9
Yoga	27	73.0
Neither Massage nor Yoga	1	2.7
Other	4	10.8

## Appendix

**Table A1.** Intervention status by primary outcome response.

Variable		Total Sample *N* = 55	Did not Respond to Survey *N* = 18	Responded to Survey *N* = 37
Intervention Status Due to COVID	Completed Trial	25 (45.4%)	3 (16.7%)	22 (59.5%)
	Paused But Returned	13 (23.6%)	1 (5.6%)	12 (32.4%)
	Paused But Did Not Return	17 (30.9%)	14 (77.8%)	3 (8.1%)

**Table A2.** Descriptive characteristics of the sample by survey response.

Variable		Total Sample *N* = 55	Did not Respond to Survey *N* = 18	Responded to Survey *N* = 37	*p* value
Age; Mean (*SD*)		42.6 (13.0)	38.2 (12.3)	44.7 (13.0)	.080
Sex; *N* (%)	Female	41 (74.5%)	14 (77.8%)	27 (73.0%)	.957
	Male	14 (25.5%)	4 (22.2%)	10 (27.0%)	
Race; *N* (%)	Asian	11 (20.0%)	3 (16.7%)	8 (21.6%)	.815
	Black	6 (10.9%)	1 (5.6%)	5 (13.5%)	
	Mixed	2 (3.6%)	1 (5.6%)	1 (2.7%)	
	Other	4 (7.3%)	1 (5.6%)	3 (8.1%)	
	White	32 (58.2%)	12 (66.7%)	20 (54.1%)	
Ethnicity; *N* (%)	Hispanic	8 (14.5%)	4 (22.2%)	4 (10.8%)	.472
	Non-Hispanic	47 (85.4%)	14 (77.8%)	33 (89.2%)	

*Note. P* values for comparisons of participant characteristics between treatment orders are obtained from independent samples *t* tests for continuous variables and Pearson chi-squared tests for categorical variables.

**Table A3.** Descriptive characteristics of the sample by pause in protocol due to COVID-19.

Variable		Total Sample *N* = 55	Paused Due to COVID (*N* = 30)	Did Not Pause Due to COVID (*N* = 25)	*p* value
Age; Mean (*SD*)		42.6 (13.0)	40.9 (13.4)	44.5 (12.6)	.313
Sex; *N* (%)	Female	41 (74.5%)	21 (70.0%)	20 (80.0%)	.591
	Male	14 (25.5%)	9 (30.0%)	5 (20.0%)	
Race; *N* (%)	Asian	11 (20.0%)	7 (23.3%)	4 (16.0%)	.665
	Black	6 (10.9%)	3 (10.0%)	3 (12.0%)	
	Mixed	2 (3.6%)	2 (6.7%)	0 (0.0%)	
	Other	4 (7.3%)	2 (6.7%)	2 (8.0%)	
	White	32 (58.2%)	16 (53.3%)	16 (64.0%)	
Ethnicity; *N* (%)	Hispanic	8 (14.5%)	6 (20.0%)	2 (8.0%)	.383
	Non-Hispanic	47 (85.4%)	24 (80.0%)	23 (92.0%)	

*Note*. *P* values for comparisons of participant characteristics between treatment orders are obtained from independent samples *t* tests for continuous variables and Pearson chi-squared tests for categorical variables.

**Table A4.** Descriptive statistics for satisfaction measures by pause in protocol due to COVID-19.

Measure		Mean (*SD*)		*p* value
		Paused Due to COVID (*N* = 30)	Did Not Pause Due to COVID (*N* = 25)	
Elements of the Personalized Trial*				
Items	1. I found the onboarding process (from the initial survey to getting my materials) for my personalized trial straightforward and easy to follow.	4.47 (0.64)	4.55 (0.51)	.680
	2. I think my Fitbit Charge 3 was easy to use.	4.47 (0.74)	4.50 (0.60)	.881
	3. The informational videos helped me understand how to participate in this study.	4.47 (0.64)	4.36 (0.90)	.706
	4. The materials I received in the mail were clear and easy to use.	4.60 (0.63)	4.64 (0.49)	.845
	5. I enjoyed receiving daily text message prompts and surveys on my cell phone.	3.87 (1.13)	3.82 (1.01)	.892
	6. I felt like I knew what was coming next in my personalized trial.	4.40 (0.63)	4.24 (0.83)	.530
	7. My personalized trial was easy to integrate into my daily routine.	4.00 (0.76)	4.27 (0.55)	.212
Satisfaction with Components of the Trial**				
Items	1. Your personalized trial of yoga and massage for chronic lower back pain.	4.67 (0.62)	4.77 (0.53)	.579
	2. Video explanations and demonstrations of study devices and procedures.	4.27 (0.88)	4.32 (0.95)	.868
	3. Text messaging for reminders.	4.20 (0.86)	4.36 (0.79)	.555
	4. Text messaging for survey questions.	4.13 (0.83)	4.41 (0.80)	.317
	5. Use of the Zeel application/website and survey for at-home yoga and massage booking.	4.27 (0.88)	4.18 (0.80)	.762
	6. Zeel as a provider for your massage therapist or yoga instructor.	4.47 (0.74)	4.45 (0.74)	.961
	7. Use of the Fitbit Charge 3 to track your activity and sleep.	4.47 (0.74)	4.55 (0.51)	.704
	8. Presentation of your results.	4.07 (1.28)	4.23 (0.87)	.652

* Questions rated on a 5-point Likert scale from 1 “Strongly Disagree” to 5 “Strongly Agree.”

** Questions rated on a 5-point Likert scale from 1 “Not at all Satisfied” to 5 “Very Satisfied.”

*Note*. *P* values are from independent samples *t* tests comparing means between the group that paused the intervention due to COVID and the group that did not.
