# Supplementary material for: A Series of Virtual Interventions for Chronic Lower Back Pain: A Feasibility Pilot Study for a Series of Personalized (N-of-1) Trials
Source: Harv Data Sci Rev. Author manuscript; Available in PMC 2023 Aug 22. (PMC10443938; doi:10.1162/99608f92.72cd8432)
Supplement: Supplementary Material [file NIHMS1881694-supplement-Supplementary_Material.docx]

*Supplemental Table 1. Intervention Status by Survey Response*

| Variable | | Total Sample  N = 55 | Did not Respond to Survey  N =18 | Responded to Survey  N=37 |
| --- | --- | --- | --- | --- |
| Intervention Status Due to COVID | Completed Trial | 25 (45.4%) | 3 (16.7%) | 22 (59.5%) |
|  | Paused But Returned | 13 (23.6%) | 1 (5.6%) | 12 (32.4%) |
|  | Paused But Did Not Return | 17 (30.9%) | 14 (77.8%) | 3 (8.1%) |

*Supplemental Table 2. Descriptive Characteristics of the Sample by Survey Response*

| Variable | | Total Sample  N = 55 | Did not Respond to Survey  N =18 | Responded to Survey  N=37 | p-value |
| --- | --- | --- | --- | --- | --- |
| Age; Mean (SD) | | 42.6 (13.0) | 38.2 (12.3) | 44.7 (13.0) | .080 |
| Sex; N (%) | Female | 41 (74.5%) | 14 (77.8%) | 27 (73.0%) | .957 |
|  | Male | 14 (25.5%) | 4 (22.2%) | 10 (27.0%) |  |
| Race; N (%) | Asian | 11 (20.0%) | 3 (16.7%) | 8 (21.6%) | .815 |
|  | Black | 6 (10.9%) | 1 (5.6%) | 5 (13.5%) |  |
|  | Mixed | 2 (3.6%) | 1 (5.6%) | 1 (2.7%) |  |
|  | Other | 4 (7.3%) | 1 (5.6%) | 3 (8.1%) |  |
|  | White | 32 (58.2%) | 12 (66.7%) | 20 (54.1%) |  |
| Ethnicity; N (%) | Hispanic | 8 (14.5%) | 4 (22.2%) | 4 (10.8%) | .472 |
|  | Non-Hispanic | 47 (85.4%) | 14 (77.8%) | 33 (89.2%) |  |

Note: p-values for comparisons of participant characteristics between treatment orders are obtained from independent samples t-tests for continuous variables and pearson chi-squared tests for categorical variables

*Supplemental Table 3. Descriptive Characteristics of the Sample by Pause in Protocol Due to COVID-19*

| Variable | | Total Sample  N = 55 | Paused Due to COVID (N=30) | Did Not Pause Due to COVID (N=25) | p-value |
| --- | --- | --- | --- | --- | --- |
| Age; Mean (SD) | | 42.6 (13.0) | 40.9 (13.4) | 44.5 (12.6) | .313 |
| Sex; N (%) | Female | 41 (74.5%) | 21 (70.0%) | 20 (80.0%) | .591 |
|  | Male | 14 (25.5%) | 9 (30.0%) | 5 (20.0%) |  |
| Race; N (%) | Asian | 11 (20.0%) | 7 (23.3%) | 4 (16.0%) | .665 |
|  | Black | 6 (10.9%) | 3 (10.0%) | 3 (12.0%) |  |
|  | Mixed | 2 (3.6%) | 2 (6.7%) | 0 (0.0%) |  |
|  | Other | 4 (7.3%) | 2 (6.7%) | 2 (8.0%) |  |
|  | White | 32 (58.2%) | 16 (53.3%) | 16 (64.0%) |  |
| Ethnicity; N (%) | Hispanic | 8 (14.5%) | 6 (20.0%) | 2 (8.0%) | .383 |
|  | Non-Hispanic | 47 (85.4%) | 24 (80.0%) | 23 (92.0%) |  |

Note: p-values for comparisons of participant characteristics between treatment orders are obtained from independent samples t-tests for continuous variables and pearson chi-squared tests for categorical variables

*Supplemental Table 4. Descriptive Statistics for Satisfaction Measures by Pause in Protocol Due to COVID-19.*

| Measure | | Mean (SD) | | p-value |
| --- | --- | --- | --- | --- |
|  |  | Paused Due to COVID (N=30) | Did Not Pause Due to COVID (N=25) |  |
| Elements of the Personalized Trial* | | | |  |
| Items | 1. I found the onboarding process (from the initial survey to getting my materials) for my personalized trial straightforward and easy to follow. | 4.47 (0.64) | 4.55 (0.51) | .680 |
|  | 2. I think my Fitbit Charge 3 was easy to use. | 4.47 (0.74) | 4.50 (0.60) | .881 |
|  | 3. The informational videos helped me understand how to participate in this study. | 4.47 (0.64) | 4.36 (0.90) | .706 |
|  | 4. The materials I received in the mail were clear and easy to use. | 4.60 (0.63) | 4.64 (0.49) | .845 |
|  | 5. I enjoyed receiving daily text message prompts and surveys on my cell phone. | 3.87 (1.13) | 3.82 (1.01) | .892 |
|  | 6. I felt like I knew what was coming next in my personalized trial. | 4.40 (0.63) | 4.24 (0.83) | .530 |
|  | 7. My personalized trial was easy to integrate into my daily routine. | 4.00 (0.76) | 4.27 (0.55) | .212 |
| Satisfaction with Components of the Trial** | | | |  |
| Items | 1. Your personalized trial of yoga and massage for chronic lower back pain. | 4.67 (0.62) | 4.77 (0.53) | .579 |
|  | 2. Video explanations and demonstrations of study devices and procedures. | 4.27 (0.88) | 4.32 (0.95) | .868 |
|  | 3. Text messaging for reminders. | 4.20 (0.86) | 4.36 (0.79) | .555 |
|  | 4. Text messaging for survey questions. | 4.13 (0.83) | 4.41 (0.80) | .317 |
|  | 5. Use of the Zeel application/website and survey for at-home yoga and massage booking. | 4.27 (0.88) | 4.18 (0.80) | .762 |
|  | 6. Zeel as a provider for your massage therapist or yoga instructor. | 4.47 (0.74) | 4.45 (0.74) | .961 |
|  | 7. Use of the Fitbit Charge 3 to track your activity and sleep. | 4.47 (0.74) | 4.55 (0.51) | .704 |
|  | 8. Presentation of your results. | 4.07 (1.28) | 4.23 (0.87) | .652 |

**Questions rated on a 5-point Likert scale from 1 “Strongly Disagree” to 5 “Strongly Agree”. **Questions rated on a 5-point Likert scale from 1 “Not at all Satisfied” to 5 “Very Satisfied”.*

*Note: p-values are from independent samples t-tests comparing means between the group which paused the intervention due to COVID and the group which did not*
